# Association of Pediatric Acute-Onset Neuropsychiatric Syndrome With Microstructural Differences in Brain Regions Detected via Diffusion-Weighted Magnetic Resonance Imaging

**DOI:** 10.1001/jamanetworkopen.2020.4063

**Published:** 2020-05-04

**Authors:** Jimmy Zheng, Jennifer Frankovich, Emily S. McKenna, Nathan C. Rowe, Sarah J. MacEachern, Nathan N. Ng, Lydia T. Tam, Peter K. Moon, Jaynelle Gao, Margo Thienemann, Nils D. Forkert, Kristen W. Yeom

**Affiliations:** 1 Stanford University School of Medicine, Stanford, California; 2 Division of Allergy, Immunology, and Rheumatology, Department of Pediatrics, Stanford University School of Medicine, Stanford, California; 3 Department of Radiology, Lucile Packard Children’s Hospital, Stanford University School of Medicine, Stanford, California; 4 Department of Radiology, University of Calgary, Calgary, Alberta, Canada; 5 Department of Pediatrics, University of Calgary, Calgary, Alberta, Canada; 6 Child and Adolescent Psychiatry and Child Development, Department of Psychiatry and Behavioral Sciences, Stanford University School of Medicine, Stanford, California

## Abstract

**Importance:**

Epidemiological studies indicate a link between obsessive-compulsive disorder and infections, particularly streptococcal pharyngitis. Pediatric acute-onset neuropsychiatric syndrome (PANS) manifests suddenly with obsessions, compulsions, and other behavioral disturbances, often after an infectious trigger. The current working model suggests a unifying inflammatory process involving the central nervous system, particularly the basal ganglia.

**Objective:**

To investigate whether diffusion-weighted magnetic resonance imaging (DWI) detects microstructural abnormalities across the brain regions of children with PANS.

**Design, Setting, and Participants:**

Case-control study performed at a single-center, multidisciplinary clinic in the United States focusing on the evaluation and treatment of children with PANS. Sixty consecutive patients who underwent 3 Tesla (T) magnetic resonance imaging (MRI) before immunomodulation from September 3, 2012, to March 30, 2018, were retrospectively reviewed for study inclusion. Six patients were excluded by blinded investigators because of imaging or motion artifacts, 3 patients for major pathologies, and 17 patients for suboptimal atlas image registration. In total, 34 patients with PANS before initiation of treatment were compared with 64 pediatric control participants.

**Main Outcomes and Measures:**

Using atlas-based MRI analysis, regional brain volume, diffusion, and cerebral blood flow were measured in the cerebral white matter, cerebral cortex, thalamus, caudate, putamen, pallidum, hippocampus, amygdala, nucleus accumbens, and brainstem. An age and sex–controlled multivariable analysis of covariance was used to compare patients with control participants.

**Results:**

This study compared 34 patients with PANS (median age, 154 months; age range, 55-251 months; 17 girls and 17 boys) and 64 pediatric control participants (median age, 139 months; age range, 48-213 months); 41 girls and 23 boys). Multivariable analysis demonstrated a statistically significant difference in MRI parameters between patients with PANS and control participants (*F*_21,74_ = 6.91; *P* < .001; partial η^2^ = 0.662). All assessed brain regions had statistically significantly increased median diffusivity compared with 64 control participants. Specifically, the deep gray matter (eg, the thalamus, basal ganglia, and amygdala) demonstrated the most profound increases in diffusivity consistent with the cardinal clinical symptoms of obsessions, compulsions, emotional dysregulation, and sleep disturbances. No statistically significant differences were found regarding volume and cerebral blood flow.

**Conclusions and Relevance:**

This study identifies cerebral microstructural differences in children with PANS in multiple brain structures, including the deep gray matter structures (eg, the thalamus, basal ganglia, and amygdala). Further study of MRI is warranted in prospective, clinical trials as a potential quantitative method for assessing patients under evaluation for PANS.

## Key Points

**Question:**

How does diffusion-weighted magnetic resonance imaging differ between patients with pediatric acute-onset neuropsychiatric syndrome and pediatric control participants?

**Findings:**

In this case-control study of 34 consecutive patients with pediatric acute-onset neuropsychiatric syndrome who had 3 Tesla magnetic resonance imaging, all assessed brain regions, particularly the deep gray matter (eg, the thalamus, basal ganglia, and amygdala), had statistically significantly increased mean diffusivity compared with 64 control participants. These diffusion abnormalities are consistent with the cardinal clinical symptoms of these patients, including obsessions, compulsions, emotional dysregulation, and sleep disturbances.

**Meaning:**

Diffusion-weighted magnetic resonance imaging may offer valuable quantitative information to assist with the diagnostic workup of pediatric acute-onset neuropsychiatric syndrome.

## Teaser

This case-control study investigates whether diffusion-weighted imaging shows microstructural abnormalities across the brain regions of children with pediatric acute-onset neuropsychiatric syndrome.

## Introduction

The diagnostic considerations of childhood-onset obsessive-compulsive disorder (OCD) have evolved substantially since the late 1980s. Early prospective studies^1,2^ of children with OCD revealed that a subgroup experienced an unusually abrupt onset (<48 hours) and a relapsing-remitting course of neuropsychiatric symptoms, notably after streptococcal pharyngitis. In parallel, research on Sydenham chorea, a neurological sequela of group A streptococcal infection, found that 50% to 70% of affected children experienced obsessions and compulsions before, during, or after onset of chorea.^3,4,5^ These findings led to the hypothesis that Sydenham chorea might share a common pathophysiology with acute-onset OCD even when chorea is absent,^5^ thus forming the basis of a new diagnostic entity, namely, pediatric autoimmune neuropsychiatric disorders associated with streptococcal infections (PANDAS).

The new PANDAS classification was subsequently met with skepticism. Concerns were raised in particular about the lack of clear distinction between PANDAS and other neuropsychiatric diagnoses, as well as the strength of the association between OCD and group A streptococcal infection.^6,7,8,9,10^ To address these issues, a group of clinicians and scientists convened in 2010 to discuss all possible cases of acute-onset OCD regardless of cause.^11^ Participants unanimously agreed that acute onset was the key distinguishing clinical feature and proposed the more inclusive term of pediatric acute-onset neuropsychiatric syndrome (PANS). Since the development of this diagnostic entity, a number of clinics and research programs have further characterized the many comorbid symptoms that can suddenly start along with OCD, including sleep disturbances, emotional dysregulation, eating restriction, pain and sensory disturbances, urinary symptoms (enuresis and polyuria), cognitive and behavior regression, anxiety, transient psychotic symptoms, oppositionality, impulsivity, irritability and rage.^12,13,14,15,16,17,18,19,20,21,22,23^

By definition, PANS includes not only PANDAS but also early, abrupt-onset OCD associated with other infectious and noninfectious triggers.^11,24^ A comprehensive diagnostic workup must be performed to rule out other known differential diagnoses, such as Sydenham chorea, Tourette syndrome, and systemic lupus erythematosus. Current recommended diagnostic testing targets inflammatory biomarkers given the link between OCD disorders and autoimmunity observed in epidemiological studies.^25,26,27,28,29,30,31^ Symptoms of PANS and PANDAS are hypothesized to result from cross-reactive antibodies that breach a compromised blood-brain barrier and damage neuronal tissues in the basal ganglia and amygdala.^32^ However, some studies^33,34^ have reported poor predictive value (17%-40% for positive predictive value and 44%-74% for negative predictive value) and low test performance (15%-60% sensitivity and 28%-92% specificity) for standard PANS diagnostic evaluations, including inflammatory markers such as erythrocyte sedimentation rate, C-reactive protein, and antibody titers. There remains a pressing need for reliable biomarkers to improve diagnostic accuracy and validate the underlying pathophysiology of PANS.

Diffusion-weighted magnetic resonance imaging (DWI) could potentially serve as a noninvasive tool for assessing microstructural differences of the brain in children with a suspected inflammatory cause of abrupt-onset OCD. Although prior research has identified volumetric and inflammatory changes in the basal ganglia,^35,36,37^ no studies to our knowledge have yet assessed cerebral blood flow (CBF), which may be associated with local inflammation, or the mean diffusivity or apparent diffusion coefficient (ADC), which measures the magnitude of water molecule diffusion within tissue. In the setting of neuroinflammation, ADC is expected to increase because of water molecules diffusing freely throughout the extracellular space.^38,39,40^ Herein, a retrospective, cross-sectional analysis was conducted of regional brain volume, ADC, and CBF differences across various brain regions in patients with PANS and in pediatric control participants. It was hypothesized that affected children would exhibit higher diffusivity compared with control participants in the deep gray matter (eg, thalamus, basal ganglia, and limbic structures) given the predominant symptoms of OCD, emotional dysregulation, and sleep disturbances. We expected limited volumetric or CBF differences because of patient group heterogeneity in the timing of imaging after an acute flare in neuropsychiatric symptoms.

## Methods

The study was approved by the Stanford University Institutional Review Board. Written informed consent was obtained from all parents, and assent was obtained for all patients in the study. A separate institutional review board protocol allowed for medical record review and analysis of magnetic resonance imaging (MRI) data under a specific protocol for the control group. This study followed the Strengthening the Reporting of Observational Studies in Epidemiology (STROBE) reporting guideline.

### Patient Selection

In this case-control study, consecutive patients evaluated in the Stanford University PANS multidisciplinary clinic from September 3, 2012, to March 30, 2018, were retrospectively reviewed. The study population was identified using the following inclusion criteria: patients were aged 4 to 18 years, met criteria for PANS (eAppendix in the Supplement), and received 3 Tesla (T) DWI before initiation of anti-inflammatory or immunomodulatory therapy. Patients with a clinical profile that better matched a diagnosis of Sydenham chorea (ie, clinically significant chorea) or Tourette syndrome (ie, waxing and waning OCD and tics) were excluded. The presence of neuropsychiatric symptoms and neurological signs at the time of MRI was investigated through review of medical records and prospectively administered patient questionnaires. The Children’s Global Assessment Scale (CGAS) is a clinician’s rating of a patient’s psychological and social function ranging from scores of 1 to 100, with higher scores indicating better functioning.^41^ Clinicians in this study received training on assigning CGAS scores and recorded scores at the end of each clinical encounter. The CGAS scores were collected in a research database, and missing scores were obtained retrospectively.

Members of the medical imaging team (E.S.M., N.D.F, and K.W.Y) who were blinded to the disease and hypothesis under study performed the image analysis and excluded images with inadequate data, such as motion artifacts and unidentified bright objects, and structural pathologies, such as masses or cysts. Among 60 consecutive patients who met study entry criteria, 6 patients were excluded by blinded investigators because of imaging or motion artifacts, 3 patients for major pathologies, and 17 patients for suboptimal atlas image registration. In total, 34 patients with PANS before initiation of treatment were compared with 64 pediatric control participants.

For the control group, all children 18 years or younger seen for evaluation via a 3T MRI system at Lucile Packard Children’s Hospital, Stanford, California, from January 5, 2010, to October 22, 2013, were retrospectively reviewed. Control participants underwent imaging as a standard of care for evaluation of syncope, nausea, family history of aneurysm or cancers, scalp nevus, cholesteatoma, sinus disease or inflammatory nasal obstruction, isolated facial lesions, orbital strabismus, or familial short stature. A thorough medical record review was performed to identify any history of systemic diseases (eg, kidney, gastrointestinal, or cardiac), cancer, prematurity, migraines, hearing loss, vascular lesions, infection, prior radiotherapy, or laboratory abnormalities. Only those individuals with normal-appearing brains on MRI and no known neurological, neurocognitive, developmental, or behavioral deficits were included. Control participants younger than 4 years were excluded to optimize age matching with the patient group. Rigorous quality control was similarly performed on control MRI. The control population has been previously described.^42^

### MRI Acquisition

Patients underwent MRI at varying points during their disease course relative to an initial or recurrent symptomatic flare-up. For all individuals, 3T MRI was obtained with an 8-channel head coil on a single MRI scanner (Discovery 750W; GE Healthcare). Pseudocontinuous arterial spin labeling (ASL) MRI was performed using the methods described by Dai et al.^43^ Briefly, this vendor-supplied ASL MRI was performed using a pseudocontinuous labeling period of 1500 milliseconds (ms), followed by a 1500-ms postlabel delay. Whole-brain images were obtained with a 3-dimensional, background-suppressed, fast spin-echo stack-of-spirals method. Multiarm spiral imaging was used, with 8 arms and 512 points obtained on each arm (bandwidth, 62.5 kHz), yielding a 3-mm^2^ in-plane spatial resolution and a 4-mm section thickness. A high level of background suppression was achieved using 4 separate inversion pulses spaced around the pseudocontinuous labeling pulse. The acquisition time was approximately 5 minutes for this sequence, which also included proton density images required for CBF quantification. For a graphic setup of the ASL, the sagittal image was used for alignment after the 3-plane localizer. Postprocessing was performed using an automated reconstruction procedure according to the microsphere methods described by Buxton et al.^44^ Other pseudocontinuous ASL MRI parameters were repetition time of 4632 ms, echo time of 10.5 ms, 24-cm field of view, and 3 excitations.

In addition, echoplanar whole-brain DWI was obtained in all patients, with repetition time of 1500 ms, echo time of 37 ms, 90° flip angle, 24 × 24–cm field of view, 128 × 128–pixel acquisition matrix interpolated to a 256 × 256–pixel matrix, 44 sections with 4-mm slice thickness, no skip, and 2 diffusion weightings of b = 0 seconds/mm^2^ and b = 1000 seconds/mm^2^, for which diffusion gradients were obtained in 3 directions and averaged. Derived from DWI, the ADC has demonstrated high reproducibility and was performed as part of routine institutional neuroimaging.^45^

### Image Processing

A custom image processing pipeline was used in this work to extract quantitative values of regional brain volume, ADC, and CBF, previously described by Forkert et al.^42^ In brief, rigid registration was used for motion correction of the DWI data set with and without diffusion weighting before calculation of the quantitative ADC parameter map using the equation described by Stejskal and Tanner.^46^ For volumetric, regional diffusion, and CBF analysis, the Montreal Neurological Institute 152 brain atlas^47^ was nonlinearly registered to the DWI data set using a concatenated affine and b-spline transformation and maximization of the mutual information metric. The resulting nonlinear transformation was used to warp the Harvard-Oxford subcortical brain regions, as defined in the Montreal Neurological Institute atlas space, to the individual-specific brain anatomy. Brain regions included in the atlas were the cerebral white matter, cerebral cortex, lateral ventricles, thalamus, caudate, putamen, pallidum, hippocampus, amygdala, nucleus accumbens, and brainstem. Three experienced, blinded observers (E.S.M., N.D.F., and K.W.Y.) performed strict quality control of all patient and control MRI registrations, excluding those with suboptimal overlap between reference and patient brain regions. The aligned brain atlas regions were then used to measure the regional brain volume, median ADC, and median CBF values for corresponding brain structures in each hemisphere. The lateral ventricles were only included in the volumetric assessment.

### Analysis of Imaging Data

Using atlas-based MRI analysis, regional brain volume, diffusion, and CBF were measured in the cerebral white matter, cerebral cortex, thalamus, caudate, putamen, pallidum, hippocampus, amygdala, nucleus accumbens, and brainstem. An age and sex–controlled multivariable analysis of covariance (MANCOVA) was used to test the null hypothesis that the mean values of MRI parameters for patients are equal to those of control participants. Only a subset of patients (n = 25) had CBF values available, so 2 separate analyses were conducted. One analysis included only volumetric and median ADC values as dependent variables, whereas the second analysis also incorporated CBF values. Both analyses used age and sex as covariates and class (patient vs control) as the fixed factor. If the outcomes of the MANCOVA were found to be statistically significant, subsequent univariate analysis was performed to identify the key MRI parameters on which patients differed from control participants. A χ^2^ test for variance was performed to assess the variability of each imaging feature in patients vs control participants given the temporal heterogeneity of cases. SPSS, version 24.0 (IBM), was used for the MANCOVA statistical analyses, and *P* < .05 was considered statistically significant after Bonferroni correction for multiple testing where applicable.

Secondary exploratory 1-way MANCOVA analyses were conducted to assess whether group differences would vary based on the presence of chorea, defined as subtle choreiform movements or twitches noted on a modified standing Romberg position or a milkmaid grip. These analyses were motivated by the initial hypothesis that Sydenham chorea and PANS share a common neuroinflammatory pathophysiology. A Bonferroni-corrected *P* < .05 was considered statistically significant after accounting for multiple-group comparisons (patients with chorea vs patients without chorea vs control participants).

In addition to the statistical analysis described above, plots with age-related 5th, 10th, 25th, 50th, 75th, 90th, and 95th quantile curves were generated for each parameter, investigated using local piecewise regression analysis described by Sakov et al^48^ based on the control group data for each brain structure and parameter. Corresponding data points for the patient group were also plotted for visual assessment. The plots were generated using the R statistical software package, version 3.2.2 (The R Foundation for Statistical Computing).

## Results

### Clinical Characteristics of Patients With PANS

**Figure 1.**
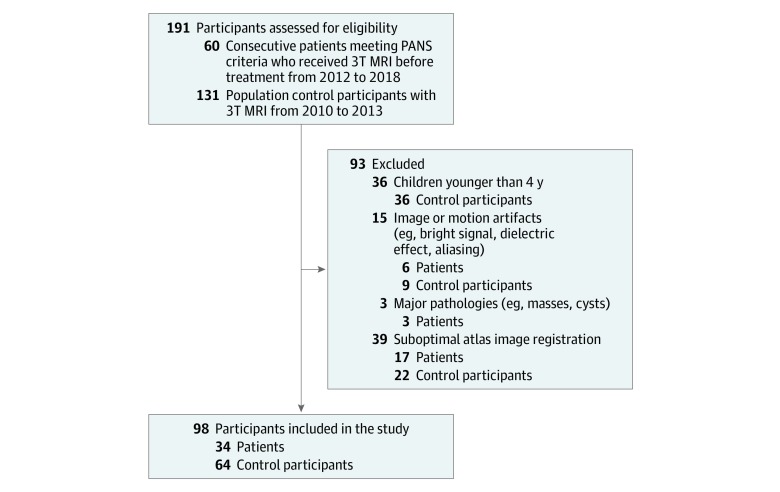
Participant Flow Diagram With Selection and Exclusion Criteria Sixty patients diagnosed as having pediatric acute-onset neuropsychiatric syndrome (PANS) and 131 control participants were selected for this case-control study. Thirty-four patients and 64 control participants were included in the final analysis. MRI indicates magnetic resonance imaging.

Included in this study were 34 patients with PANS (median age, 154 months; range, 55-251 months) and 64 pediatric control participants (median age, 139 months; range, 48-213 months). The patient group consisted of 17 girls and 17 boys; the control group consisted of 41 girls and 23 boys. Twelve patients (35%) were classified as having new acute-onset psychiatric symptoms, 7 (21%) as having chronic static psychiatric symptoms since PANS onset, and 15 (44%) as having an acute flare with chronic symptoms (Table 1).^41^ Patients underwent MRI at different time points after symptomatic presentation, with patients with new acute-onset psychiatric symptoms receiving imaging within days of initial onset and some patients receiving imaging years after initial onset (mean time after initial onset, 404 days; range, 9 days for an acute-onset case to 4097 days for a chronic case). The detailed selection process, including patient and control inclusion and exclusion criteria, is shown in Figure 1.

**Table 1.** Clinical Characteristics Evaluated at the Time of Magnetic Resonance Imaging (MRI) Among 34 Patients With Pediatric Acute-Onset Neuropsychiatric Syndrome Included in the Study

Variable	No. (%) (n = 34)
Age at initial neuropsychiatric decline, mean (SD), y	10.4 (3.1)
Age at MRI date, mean (SD), y	12.9 (3.9)
CGAS score, mean (SD)^a^	49.1 (15.3)
Disease status at MRI	
New acute-onset psychiatric symptoms	12 (35)
Chronic static psychiatric symptoms	7 (21)
Acute flare with chronic symptoms	15 (44)
Neuropsychiatric symptoms at the time of MRI	
Obsessions and compulsions	33 (97)
Eating restriction	16 (47)
Anxiety	23 (68)
Emotional dysregulation	27 (79)
Irritability or rage	21 (62)
Oppositionality	7 (21)
Hyperactivity or impulsivity	8 (24)
Attention issues	12 (35)
Behavior regression	7 (21)
Cognitive difficulties	14 (41)
Urinary symptoms of enuresis or polyuria	8 (24)
Temperature dysregulation	3 (9)
Sensory amplification	16 (47)
Sleep disturbance	23 (68)
Psychosis, including hallucinations and/or delusions and/or catatonia	6 (18)
Tics	14 (41)
Waking unrefreshed and daytime fatigue, total score ≥3	16 (47)
Pain dysregulation in ≥3 areas	7 (21)
Neurological examination at the time of MRI	
Positive glabellar tap reflex	9 (26)
Tongue fasciculations or wormian tongue, but not darting	12 (35)
Truncal instability or slumped posture	19 (56)
Overflow dystonia on straight arm raise	4 (12)
Overflow dystonia on stress gait	4 (12)
Subtle choreiform movements or twitches, but not tics^b^	18 (53)

Abbreviation: CGAS, Children’s Global Assessment Scale.

^a^ The CGAS is a rating of a child’s psychological and social functioning. The score ranges from 1 to 100 and is based on clinician assessment.^41^

^b^ In all of these patients, the psychiatric symptoms were prominent and severe, but the subtle choreiform movements or twitches in limbs or hands seen with the Romberg test were subtle, infrequent, and not noticed by the patient, family, or referring clinician. Clinically apparent cases of chorea that better fit the diagnosis of Sydenham chorea were excluded from this study.

All patients had an abrupt onset or relapse of obsessions and compulsions and had at least 2 other severe behavioral or psychiatric symptoms meeting PANS criteria. For the 14 patients (41%) with tics, the psychiatry team (including M.T.) excluded the possibility of Tourette syndrome because the illness onset and psychiatric comorbidities more closely fit the PANS criteria. Eighteen patients (53%) had findings of subtle choreiform movements or twitches on the Romberg test. These results were not clinically significant because they were not noticed by the patient, family, or referring clinician. Given that patients did not have frank chorea and that the impairing symptoms were predominantly psychiatric, no patient was referred to neurology for evaluation of chorea. Only 14 patients (41%) had evidence of a preceding group A streptococcal infection. Children’s Global Assessment Scale scores (range, 1-100), obtained on average 6 to 7 days before MRI, indicated severe psychiatric impairment (mean [SD] score, 49.1 [15.3]).

### Group Comparison Between PANS and Control

The 1-way MANCOVA analysis among all 34 patients (with age and sex as covariates) demonstrated a statistically significant group difference between patients with PANS and control participants (*F*_21,74_ = 6.91; *P* < .001; partial η^2^ = 0.662). Univariate analysis showed that all ADC values were statistically significantly increased in the brain structures analyzed, particularly the thalamus (*F*_1,96_ = 67.38; *P* < .001), caudate (*F*_1,96_ = 41.10; *P* < .001), putamen (*F*_1,96_ = 40.87; *P* < .001), pallidum (*F*_1,96_ = 46.79; *P* < .001), and amygdala (*F*_1,96_ = 27.31; *P* < .001). No difference regarding any volume measurement (*F*_1,96_<2.41; *P* > 1.24) was found between the groups (Table 2). However, the volume variability of all brain regions, except for the caudate, putamen, hippocampus, and nucleus accumbens, was larger in patients vs control participants, although the differences did not reach statistical significance (*t*_33_<39.45; *P* > .204). The separate MANCOVA analysis among the 25 patients with suitable CBF data (age and sex as covariates) also showed a statistically significant group difference (*F*_31,55_ = 4.57; *P* < .001; partial η^2^ = 0.720). All ADC values were statistically significantly increased in patients vs control participants (*F*_1,87_ > 8.08; *P* < .006). Univariate analysis showed limited differences in CBF between groups, with only the cerebral cortex reaching statistical significance (*F*_1,87_ = 4.02; *P* = .048). Variability in CBF to all brain regions was larger in patients vs control participants, but no differences reached statistical significance (*t*_24_<25.75; *P* > .366).

**Table 2.** Volumetric, Apparent Diffusion Coefficient (ADC), and Cerebral Blood Flow (CBF) Analyses by Brain Region

Variable	Mean (SE) [95% CI]^a^			Univariate test		
	PANS (n = 34)	Control (n = 64)		Mean difference (PANS minus control)	*F* distribution	*P* value^b^
Regional brain volume, mL						
Cerebral white matter	195.12 (3.21) [188.75-201.49]	196.42 (2.32) [191.81-201.03]	−1.30		0.11	.75
Cerebral cortex	365.74 (6.02) [353.79-377.69]	365.96 (4.36) [357.31-374.62]	−0.22		0.00	.98
Lateral ventricles	5.91 (0.11) [5.70-6.12]	5.98 (0.08) [5.83-6.13]	−0.07		0.29	.59
Thalamus	6.88 (0.11) [6.66-7.10]	6.92 (0.08) [6.76-7.08]	−0.04		0.09	.76
Caudate	2.84 (0.05) [2.74-2.95]	2.85 (0.04) [2.77-2.92]	−0.01		0.01	.94
Putamen	4.78 (0.08) [4.61-4.94]	4.72 (0.06) [4.60-4.84]	0.06		0.33	.57
Pallidum	1.52 (0.03) [1.47-1.58]	1.53 (0.02) [1.49-1.57]	−0.01		0.03	.86
Hippocampus	3.30 (0.05) [3.19-3.41]	3.26 (0.04) [3.18-3.34]	0.04		0.37	.54
Amygdala	1.66 (0.03) [1.60-1.72]	1.62 (0.02) [1.57-1.66]	0.04		1.01	.32
Nucleus accumbens	0.41 (0.01) [0.39-0.43]	0.39 (0.01) [0.38-0.41]	0.02		1.33	.25
Brainstem	23.50 (0.39) [22.72-24.28]	24.26 (0.28) [23.70-24.82]	−0.76		2.41	.12
Median ADC, 10^−6^ mm^2^/s						
Cerebral white matter	854.44 (4.52) [845.46-863.41]	818.75 (3.27) [812.25-825.25]	35.69		40.12	<.001^c^
Cerebral cortex	894.38 (3.59) [887.25-901.51]	874.55 (2.60) [869.39-879.71]	19.83		19.63	<.001^c^
Thalamus	874.33 (7.65) [859.13-889.52]	796.04 (5.54) [785.03-807.05]	78.29		67.38	<.001^c^
Caudate	849.78 (7.38) [835.13-864.44]	790.82 (5.34) [780.21-801.44]	58.96		41.10	<.001^c^
Putamen	834.84 (7.13) [820.68-848.99]	778.05 (5.16) [767.80-788.30]	56.79		40.87	<.001^c^
Pallidum	913.85 (10.98) [892.06-935.65]	820.29 (7.95) [804.50-836.07]	93.56		46.79	<.001^c^
Hippocampus	951.21 (5.95) [939.39-963.04]	919.91 (4.31) [911.34-928.47]	31.30		17.80	<.001^c^
Amygdala	910.61 (7.42) [895.88-925.35]	862.29 (5.37) [851.62-872.96]	48.32		27.31	<.001^c^
Nucleus accumbens	873.81 (9.43) [855.10-892.53]	820.99 (6.83) [807.43-834.54]	52.82		20.23	<.001^c^
Brainstem	809.48 (4.71) [800.12-818.84]	784.63 (3.41) [777.85-791.41]	24.85		17.88	<.001^c^
Median CBF, mL/100 g/min^d^						
Cerebral white matter	45.71 (1.55) [42.62-48.80]	47.57 (0.94) [45.69-49.44]	−1.86		0.99	.32
Cerebral cortex	61.53 (2.28) [57.00-66.07]	67.03 (1.38) [64.28-69.78]	−5.50		4.02	.048^c^
Thalamus	54.24 (2.18) [49.92-58.57]	55.76 (1.32) [53.14-58.38]	−1.52		0.33	.56
Caudate	51.21 (1.66) [47.91-54.51]	54.64 (1.01) [52.64-56.64]	−3.43		2.97	.09
Putamen	53.15 (1.69) [49.80-56.51]	56.57 (1.02) [54.54-58.60]	−3.42		2.84	.10
Pallidum	43.06 (1.63) [39.83-46.29]	44.06 (0.99) [42.10-46.01]	−1.00		0.26	.61
Hippocampus	53.84 (1.92) [50.02-57.65]	54.40 (1.16) [52.09-56.71]	−0.56		0.06	.81
Amygdala	47.97 (1.79) [44.42-51.53]	51.08 (1.08) [48.92-53.23]	−3.11		2.08	.15
Nucleus accumbens	57.77 (1.90) [54.00-61.54]	58.26 (1.15) [55.98-60.55]	−0.49		0.05	.83
Brainstem	49.62 (2.03) [45.57-53.66]	46.35 (1.23) [43.90-48.80]	3.27		1.79	.19

Abbreviation: PANS, pediatric acute-onset neuropsychiatric syndrome.

^a^ Covariates in the model are evaluated at the following values: age 143.88 months and sex 0.59.

^b^ *P* values are based on the linearly independent pairwise comparisons among the estimated marginal means (Bonferroni corrected).

^c^ *P* < .05.

^d^ Analysis was conducted on a patient subset (n = 25) because of limited data. Covariates in the model are evaluated at the following values: age 146.20 months and sex 0.56.

To assess whether patients with subtle choreiform movements or twitches differed from those without these movements, 3 secondary MANCOVA analyses were performed with regional brain volume, median ADC, and median CBF parameters as dependent variables, respectively. Statistically significant differences in each analysis were detected between patients with PANS with or without subtle choreiform movements or twitches and control participants (*F*_20,150_<2.17; *P* < .005). No statistically significant differences were found comparing these 2 PANS subgroups. Exploratory post hoc pairwise analyses comparing patients with PANS with subtle choreiform movements or twitches vs control participants showed statistically significant differences in all ADC values (minimal mean [SD] difference, −26.00 [5.47]; *P* < .001), consistent with previous results. The subgroup without these movements demonstrated statistically significant differences in ADC values only for cerebral white matter, thalamus, caudate, putamen, pallidum, and amygdala (minimal mean [SD] difference, 30.39 [11.94]; *P* < .04). These differences were also smaller in magnitude compared with those between patients with PANS with subtle choreiform movements or twitches and control participants. No differences in the univariate regional brain volume and CBF analyses reached statistical significance.

### Qualitative Analysis

**Figure 2.**
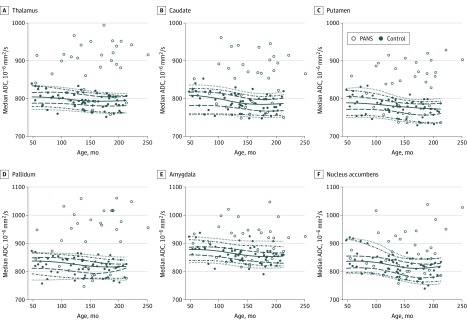
Visual Analysis of the Median Apparent Diffusion Coefficient (ADC) Regression Analysis for Patients With Pediatric Acute-Onset Neuropsychiatric Syndrome (PANS) and Control Participants A-F, Control participants are indicated by the solid circles, with corresponding age-related 5th, 10th, 25th, 50th, 75th, 90th, and 95th quantile curves based on local piecewise regression analysis. Data points for patients are indicated by the open circles for visual comparison.

The results of the statistical evaluation were also confirmed by visual analysis of the plots generated using local piecewise regression analysis. Overall, no difference between patients with PANS and control participants is obvious for regional brain volume or CBF values analyzed (eFigure in the Supplement). However, the plots clearly show statistically significantly increased ADC values across brain regions, especially in the deep gray matter (thalamus, basal ganglia, amygdala) and nucleus accumbens (Figure 2).

## Discussion

It was expected that patients with PANS would exhibit increased regional diffusion across thalamic, basal ganglia, and limbic structures compared with control participants given the hypothesized inflammatory pathophysiology and cardinal psychiatric symptoms.^37,49,50^ This study identified increased diffusion patterns across patients in all brain regions analyzed, with the most pronounced differences in the deep gray matter, including the thalamus, basal ganglia (caudate, putamen, and pallidum), and amygdala. Clinically, the most affected areas align with the type of neuropsychiatric symptoms seen in PANS. Inflammatory changes to the amygdala may impair emotional regulation, leading to sudden-onset emotional lability and irritability.^50,51,52^ Basal ganglia disruption may result in motor dysregulation and obsessive-compulsive symptoms.^53^ Thalamic abnormalities are associated with these alterations given its central role in mediating interconnectivity with multiple regions, such as the basal ganglia and amygdala,^54,55^ as well as its influence on sleep-wake cycles.^56^

Prior studies^35,36^ of MRI in patients with recent-onset PANDAS and Sydenham chorea noted increased volume in the basal ganglia during acute presentation compared with control participants. Anecdotal evidence has also shown that basal ganglia size might correlate with the disease course because striatal volume tends to normalize during remission.^57^ In the present study, no volumetric differences were found between patients with PANS and control participants, likely because most patients were in a chronic disease state (Table 1), unlike in prior studies. The patient distribution across the disease states may have diluted the influence of transient volumetric changes seen in recent-onset disease, resulting in a wider variability of brain region volumes in patients compared with control participants. Additional studies enrolling patients in similar disease states are needed to validate volumetric patterns for PANS.

No statistically significant group differences were found in CBF, but a larger variability in CBF values was observed among patients with PANS, likely because of the patient distribution across disease states. Changes in CBF often reflect a physiological response to local or systemic neuroinflammation.^38^ Therefore, with a larger patient sample and longitudinal follow-up imaging, we might see an initial acute increase in CBF with a subsequent, long-term decline as a self-limiting reaction to the inflammation. Based on the patients in this study, statistically significantly elevated ADC values in the absence of CBF differences suggest that CBF changes may not play a direct role in mediating neuropsychiatric symptoms. Furthermore, separate MANCOVA analyses with and without CBF data generated similar results, indicating that the sample size included in our study was suitable for statistical analysis.

To our knowledge, this study is the first to describe diffusion MRI–based microstructural differences in patients with PANS. Some clinicians may argue that the patients with subtle choreiform movements or twitches may actually have Sydenham chorea. These extremity movements may alternatively represent the choreiform syndrome initially reported in 1962 by Prechtl and Stemmer,^58^ which is present in up to 38% to 47% of children with severe psychiatric or behavioral disorders.^59^ Ultimately, we classified the patients in this study as having PANS because their disabling symptoms were prominently psychiatric, with only subtle choreiform movements or twitches. Although the underlying neuroinflammatory response in PANS and Sydenham chorea has not yet been established, multivariable analysis herein demonstrated that brain MRI for patients with similar psychiatric profiles did not differ statistically significantly when subclassified by the presence of subtle choreiform movements or twitches. However, post hoc analyses for individual ADC values identified statistically significant diffusion abnormalities in more brain regions for patients with subtle choreiform movements or twitches. This finding may provide support for the hypothesis that PANS and Sydenham chorea are on the same clinical spectrum, in which patients with chorea experience more diffuse inflammation. Larger, higher-powered studies are required to confirm these results and to distinguish patients with PANS from those with phenotypically similar disorders, such as pediatric OCD, that are well described in the neuroimaging literature.^60,61,62^

Because of skepticism around the inflammatory cause of PANS, a recent commentary recommends against laboratory or neuroimaging tests in children with mild to moderate, acute-onset, psychiatric-only presentations.^63^ However, our MRI findings revealed substantial cerebral diffusion abnormalities among patients with and without subtle choreiform movements or twitches, as well as across a wide range of CGAS scores, a validated measure of impairment.^41^ Further research should consider leveraging advanced regression models to understand which MRI parameters lead to the largest improvements in diagnostic accuracy for PANS. Given natural variation in MRI quality and atlas registration, future studies may also explore less rigid quality control restrictions in the patient selection process to expand the addressable population.

### Limitations

Our study has several limitations. Because this investigation was a retrospective study, MRI was obtained at different time points in the disease course, so the high number of patients with chronic symptoms in this study may have introduced bias. However, all patients selected were homogeneous in having abrupt onset or relapse of obsessions and compulsions in childhood and were experiencing disabling psychiatric symptoms at the time of MRI. Furthermore, this study included a broad range of CGAS scores in the patient sample, suggesting notable diffusion abnormalities across the spectrum of disease severity. However, this metric only considers a child’s global functioning over the past week and does not take into account the child’s baseline function before PANS. Statistically, we also note that the effect size or partial η^2^ of the MANCOVA tests may be considered large based on benchmarks in the literature,^64^ but these measures will need to be reassessed with future MRI studies.^65^

In addition, radiological findings herein may have been biased by the variable time from last flare onset to MRI acquisition. The influence of disease state on cerebral diffusion changes is unknown. For the present analysis, the well-established adult Montreal Neurological Institute atlas was registered to the MRI of patients with PANS and control participants. Alternatively, age-appropriate atlases for pediatric brains could have been more appropriate, but results may not be directly comparable with the use of multiple atlases.^66^ Another limitation of this study is the omission of trends in laterality given that data were merged from both the left and right hemispheres to reduce the number of hypotheses tested. However, lateral differences were not expected in PANS because the underlying inflammatory pathophysiology is not hypothesized to target a specific hemisphere. Finally, although segmentation of small cerebral structures may be imperfect, an automated approach vetted by visual quality control was used to ensure reproducibility and minimize the observer bias common in manual segmentation.^67,68,69^

## Conclusions

This study identifies cerebral microstructural differences in children with acute-onset OCD manifesting as PANS compared with control participants. The hypothesis that neuroinflammation is the underlying cause of acute-onset OCD in PANS may explain the MRI diffusion differences in multiple brain structures observed herein, particularly the deep gray matter structures, such as the thalamus, basal ganglia, and amygdala. Further study of MRI is warranted in prospective clinical trials as a potential tool to quantitatively assess pediatric patients who are under evaluation for PANS.

## Notes

Supplement. **eAppendix.** Criteria for Diagnosis of PANS **eFigure.** Visual Analysis of Volumetric Regression Analysis for Healthy Controls and Patients With PANS **eReference.**
