# Supplementary material for: Association of Pediatric Acute-Onset Neuropsychiatric Syndrome With Microstructural Differences in Brain Regions Detected via Diffusion-Weighted Magnetic Resonance Imaging
Source: JAMA Netw Open. 2020 May 4;3(5):e204063. doi: 10.1001/jamanetworkopen.2020.4063 (PMC7199120; doi:10.1001/jamanetworkopen.2020.4063)
Supplement: Supplement. — eAppendix. Criteria for Diagnosis of PANS eFigure. Visual Analysis of Volumetric Regression Analysis for Healthy Controls and Patients With PANS eReference. [file jamanetwopen-3-e204063-s001.pdf]

Supplementary Online Content

Zheng J, Frankovich J, McKenna ES, et al. Association of pediatric acute-onset neuropsychiatric syndrome with microstructural differences in brain regions detected via diffusion-weighted magnetic resonance imaging. *JAMA Netw Open*. 2020;3(5):e204063. doi:10.1001/jamanetworkopen.2020.4063

eAppendix. Criteria for Diagnosis of PANS

eFigure. Visual Analysis of Volumetric Regression Analysis for Healthy Controls and Patients With PANS

eReference.

This supplementary material has been provided by the authors to give readers additional information about their work.

© 2020 Zheng J et al. *JAMA Network Open*.

eAppendix. Criteria for Diagnosis of PANS[1]

1. Abrupt, dramatic onset of obsessive-compulsive disorder or severely restricted food intake
2. Concurrent presence of additional neuropsychiatric symptoms, with similarly severe and acute onset, from at least two of the following seven categories:
    a. Anxiety
    b. Emotional lability and/or depression
    c. Irritability, aggression and/or severely oppositional behaviors
    d. Behavioral (developmental) regression
    e. Deterioration in school performance
    f. Sensory or motor abnormalities
    g. Somatic signs and symptoms, including sleep disturbances, enuresis or urinary frequency
3. Symptoms are not better explained by a known neurologic or medical disorder, such as Sydenham chorea, systemic lupus erythematosus, Tourette disorder or others.

© 2020 Zheng J et al. *JAMA Network Open*.

eFigure. Visual Analysis of Volumetric Regression Analysis for Healthy Controls and Patients With PANS

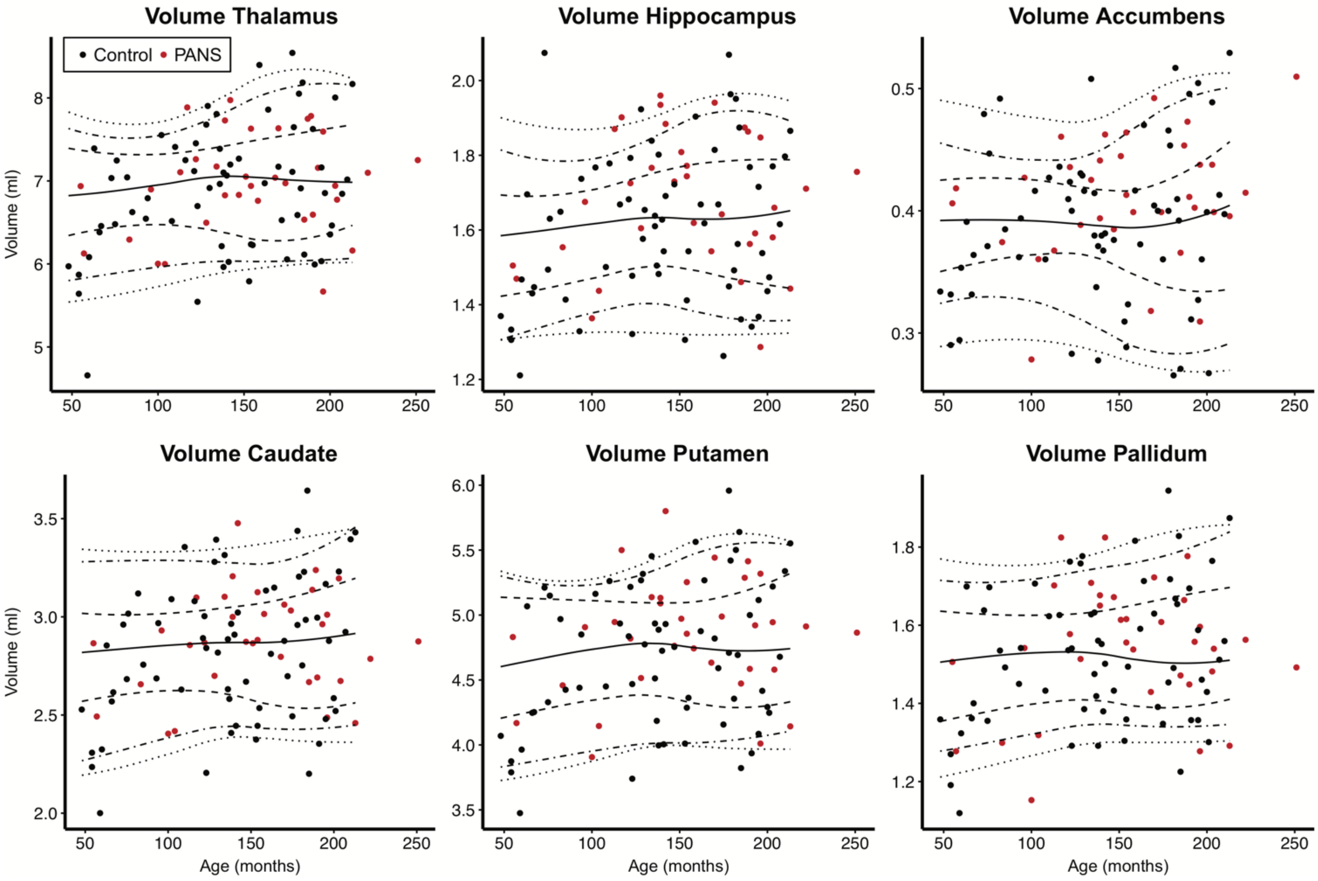

Caption: Controls are indicated by the black circles with corresponding age-related 5th, 10th, 25th, 50th, 75th, 90th, and 95th quantile curves based on local piecewise regression analysis. Data points for patients are shown by the red circles for visual comparison.

© 2020 Zheng J et al. *JAMA Network Open*.

eReference.

1. Swedo SE, Leckman JF, Rose NR. From Research Subgroup to Clinical Syndrome: Modifying the PANDAS Criteria to Describe PANS (Pediatric Acute-onset Neuropsychiatric Syndrome). *Pediatrics & Therapeutics*. 2012;2(2):1-8. doi:10.4172/2161-0665.1000113

© 2020 Zheng J et al. *JAMA Network Open*.
